# Walking with increased step length variability increases the metabolic cost of walking in young adults

**DOI:** 10.1242/jeb.250126

**Published:** 2025-04-24

**Authors:** Adam B. Grimmitt, Maeve E. Whelan, Douglas N. Martini, Wouter Hoogkamer

**Affiliations:** Department of Kinesiology, University of Massachusetts Amherst, Amherst, MA 01003, USA

**Keywords:** Energetics, Step frequency, Gait variability, Locomotion, Visually guided stepping

## Abstract

Several studies have observed a relationship between step length variability and the metabolic cost of walking. In those studies, changes in step length variability were secondary to changes in walking speed or step width variability. The purpose of this study was to determine how directly increasing step length variability affects the metabolic cost of walking. Eighteen healthy young adults completed 5 min trials of treadmill walking at 1.20 m s^−1^ while we manipulated their step length variability. Illuminated rectangles were projected onto the surface of a treadmill to cue step length variabilities of 0%, 5% and 10% coefficient of variation. Step length and its variability were tracked with reflective markers on the feet. Metabolic power across habitual (no projections) and the three variability conditions was measured using indirect calorimetry and analyzed using linear mixed effects modeling. Metabolic power was largest in the 10% condition (mean±s.d. 4.30±0.23 W kg^−1^) compared with 0% (4.16±0.18 W kg^−1^) and habitual (3.98±0.25 W kg^−1^). Actual step length variability was significantly different from prescribed conditions: 0%, 3.17±0.64%; 5%, 4.38±0.98% and 10%, 6.94±1.07%. For every 1% increase in step length variability, there was a 1.1% (0.05 W kg^−1^; *P*<0.001) increase in metabolic power. Our results demonstrate an association between the metabolic cost of walking and step length variability. This suggests that increased gait variability contributes to a small portion of the increased cost of walking seen in older adults and people with neurological impairments.

## Teaser

**Summary:** For every 1% increase in step length variability, there is a 1.1% increase in metabolic power. This suggests that step length variability contributes modestly to the cost of human walking.

## INTRODUCTION

With age and neurological impairment, people walk slower (Bohannon, 1997; Steffen et al., 2002), with shorter steps (Osoba et al., 2019), greater gait variability (Owings and Grabiner, 2004) and increased metabolic energy demands (Christiansen et al., 2009; Martin et al., 1992; Zamparo et al., 1995). Increased metabolic cost of walking has been mentioned as a potential risk factor for reduced gait speed and mobility in older individuals (Schrack et al., 2012). Reduced gait speed is associated with mortality (Newman et al., 2006; Studenski et al., 2011), cardiovascular disease and other adverse effects (Abellan van Kan et al., 2009; Szanton et al., 2021). As outlined by Boyer et al. (2023): ‘… the significant societal, economic, and personal burdens associated with mobility limitations highlight the importance of understanding the mechanisms for increased metabolic cost of walking…’. While the association between reduced gait speed and increased metabolic cost is well studied (but not yet fully understood; Boyer et al., 2023), evidence of gait variability – specifically step length variability – impacting the metabolic cost of walking independent of changes in speed is lacking.

Regardless of their age or neurological impairment, people adjust both step length and step frequency to optimize their gait. At a set speed, stride frequency and stride length are inversely proportional to one another (Zarrugh et al., 1974), and manipulation of one will result in reciprocal changes in the other. Indeed, at a constant speed, people habitually self-select a step frequency and step length that minimizes metabolic cost (Holt et al., 1995; Zarrugh and Radcliffe, 1978). To better understand the determinants of the metabolic cost of walking, researchers have manipulated stride length or stride frequency during walking. In the past, modifying step frequency was common (implemented easily using a metronome). This work determined that reducing stride length from habitual, by increasing stride frequency by 15 strides per minute, increases metabolic cost by approximately 19% during walking (Holt et al., 1991). As step frequency increases, step length decreases and the metabolic cost of moving the legs increases (Doke et al., 2005). As stride frequency decreases, i.e. as stride length increases, from habitual by 15 strides per minute, metabolic cost increases by almost 30% (Holt et al., 1991), likely resulting from the increased metabolic cost of redirecting the center of mass between steps (Donelan et al., 2002). These findings indicate an increase in metabolic cost when stride length (and stride frequency) changes from habitual.

People rarely walk with consistent step lengths in daily life, as walking often occurs in short bouts (Seethapathi and Srinivasan, 2015), at variable speeds and not on level ground (Kowalsky et al., 2021; Voloshina et al., 2013). Varying step length can also be helpful when encountering obstacles (Patla et al., 1991) or maintaining stability (McAndrew Young and Dingwell, 2012). Even for a long walking bout, at a constant speed, on level ground, with one's optimal average step length, there will be small variabilities in step length around this average – a mix of both steps that are shorter and steps that are longer than optimal (Owings and Grabiner, 2004) – due to intrinsic sensory and neuromotor noise (Dean et al., 2007; Dingwell et al., 2017). Added variability can be expected to increase metabolic cost (O'Connor et al., 2012), as each step deviates from optimal step parameters (Holt et al., 1995).

Few studies have tried to quantify the impact of step length variability on metabolic cost (O'Connor et al., 2012; Rock et al., 2018). O'Connor et al. (2012) used virtual visual flow field perturbations to increase gait variability and evaluate metabolic power. At a constant speed of 1.25 m s^−1^, high-frequency medio-lateral rotations of the virtual visual flow field induced the largest increase in metabolic power (5.9%). Participants walked with increased step length variability in response to the perturbation, but the metabolic increase was most strongly coupled with increased step width variability. More recently, Rock et al. (2018) evaluated metabolic cost of transport and step length variability across a range of walking speeds. Although speed had the greatest impact on metabolic cost, at a constant speed of 1.25 m s^−1^, each 1% increase in step length variability was also associated with a 5.9% increase in metabolic cost. In these studies, the observed increases in metabolic cost with increased step length variability could not be separated from changes in step width variability or walking speed, respectively, so the isolated effects of increased step length variability on the metabolic cost of walking are still unknown.

Therefore, we set out to quantify the isolated effects of increased step length variability on the metabolic cost of walking. We projected illuminated rectangular visual cues (stepping stones) onto a treadmill to increase step length variability (Hollands et al., 1995; Hoogkamer et al., 2015; Roerdink et al., 2009; Van Ooijen et al., 2015). The stepping stones progressed at the same speed as the treadmill and were spaced to the participant's habitual step length, with increasing levels of step length variability per condition. By directly targeting step length variability, using projected stepping stones, we were able to evaluate the metabolic cost of walking with increased step length variability independent from other gait changes that may arise when using perturbations that indirectly affect step length variability. We hypothesized that increases in step length variability would increase the metabolic cost of walking.

## MATERIALS AND METHODS

### Participants

Eighteen healthy young adults (7 women and 11 men; mean±s.d. age 24.4±3.7 years, height 171.2±17.2 cm, mass 70.5±13.3 kg) completed this study. Sample size was determined using a convenience sample that was similar to other studies within the literature (Rock et al., 2018: *n*=10; O'Connor et al., 2012: *n*=11). Eligible participants were between the ages of 18 and 45 years old, had not experienced lower extremity injuries or surgery within the past 6 months, and were free of any existing orthopedic, cardiovascular or neuromuscular conditions. Written informed consent was obtained from each participant prior to the study. All procedures were approved by the Institutional Review Board at the University of Massachusetts Amherst (#3002).

### Procedures

We provided each participant with a pair of standardized shoes in their size (Speed Sutamina, PUMA SE, Herzogenaurach, Germany). We placed retroreflective markers on each foot at the fifth metatarsal head and calcaneus, and a four-marker cluster on the sacrum. Participants first walked at a speed of 1.20 m s^−1^ (Das Gupta et al., 2019) for 5 min to familiarize themselves with the dual-belt treadmill (Bertec, Columbus, OH, USA) and the indirect calorimetry mouthpiece. Next, they walked for 3 min at 1.20 m s^−1^ for which we evaluated habitual step length and width during the final 30 s. An eight-camera Miqus system (Qualisys, Gothenburg, Sweden) recorded kinematic data at 100 Hz. We used kinematic data from the left and right calcaneus to determine average habitual step length and step width using a custom Matlab script (The MathWorks, Natick, MA, USA). Step length for each leg was calculated as the distance between the anterior and posterior positions of the ipsilateral and contralateral calcaneus markers, respectively, during the maximum anterior position of the calcaneus marker for each step (Desailly et al., 2009). Step width was calculated for each leg during midstance as the mediolateral distance between the stance leg calcaneus marker and the contralateral marker during the subsequent midstance. Mean step length and standard deviation were used to determine the coefficient of variation, i.e. the standard deviation divided by the mean step length for each foot, before averaging across feet.

Experimental conditions were: no projections (NP), 0%, 5% and 10% variability. Throughout, we consider step length variability in terms of the coefficient of variation of the step length during a trial. At 0% variability, we projected stepping stones with no variability in step length, whereas for the 10% condition we projected stepping stones with 10% variability in step length across the entire trial. The projected stepping stones were medio-laterally spaced to habitual step width. In a block randomized order, participants completed all four experimental conditions, before completing them again in reverse, for a total of eight trials (e.g. NP, 10%, 0%, 5%, 5%, 0%, 10%, NP). Participants were informed that they would be walking in a condition with random step length variability but were not told whether the condition would be 0%, 5% or 10% variability. They were instructed to try and hit the center of the stepping stone with the center of their foot, and not to go back and step on a stepping stone should they miss it the first time.

**Fig. 1.**
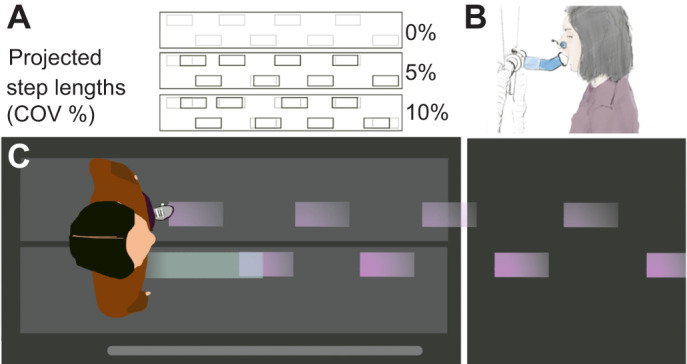
**Experimental conditions and setup.** (A) Diagram of example step length conditions for 0%, 5% and 10% variability (COV, coefficient of variation). Gray rectangles are stepping stone targets projected at a participant's specific habitual step length. Black rectangles are the lengthwise deviations of perturbed stepping stones generated randomly from discretized bins. (B) Illustration of indirect calorimetry including the pitched 45 deg extension used to open the participant’s field of view. (C) Top-down visual of a participant targeting projected stepping stones (purple; moving from right to left) for 0% variability.

The projected stepping stones were generated using a custom Matlab script. A vector of step lengths for the entire trial was created based on the participant's habitual step length, treadmill speed (1.20 m s^−1^) and trial duration (5 min). A vector of corresponding step length perturbations was created using the *randn* function in Matlab, which generates a list of normally distributed random numbers with a mean of 0 and variance of 1. This step length perturbation vector was scaled by the desired step length variability (0%, 5% or 10% coefficient of variation) and the habitual step length. To ensure that differences in step length were perceivable by the participants, we assigned each vector value into bins that were 5% multiples of the participant's habitual step length. We limited perturbations to a maximum of 15% in either direction, to prevent the possibility of unrealistic perturbations at the tails of the normal distribution. Perturbations that fell within half of the discretization interval above or below a perturbation target were reassigned to the target value and applied. Participants encountered distances that matched their habitual step length most of the time, with less frequent longer and shorter perturbed steps (Fig. 1A), and a larger coefficient of variation increased the average magnitude of a perturbed step.

We used an expired-gas analysis system (True One 2400, Parvo Medics, Salt Lake City, UT, USA) to measure metabolic power across the four experimental walking conditions. We added a 3D printed extension to the two-way valve mouthpiece (Hans Rudolf Mouthpiece, Shawnee, KS, USA) that pitched forward (5 cm) and up (5 cm) at a 45 deg angle (Fig. 1B). The participants were then able to see the approaching stepping stones. While participants were walking without projections (NP), we instructed them to look downwards towards the front of the treadmill. Each trial was 5 min long and data across the last 2 min of each trial were used to evaluate metabolic power. A 5 min rest period was provided at the end of each trial. We calculated metabolic power (W kg^−1^) using oxygen uptake, carbon dioxide production, and the Péronnet and Massicotte equation (Kipp et al., 2018; Péronnet and Massicotte, 1991). Metabolic power was not normalized to baseline walking or standing. Instead, random intercepts for each subject were applied within a linear mixed effects model.

### Statistical analysis

We used a linear mixed effects model to evaluate changes in metabolic power with increasing step length variability. We used the actual step length variability that participants walked with for each condition, rather than the projected step length variability for that trial. All statistical analysis was performed in RStudio (4.2.2) with a linear mixed effects regression (Wilkinson et al., 2023) using lme4 (1.1-32) and sjstats (0.18.2) packages. A paired *t*-test was used to compare metabolic power and step length variability between the NP and 0% conditions. A linear model was used to investigate the impact of step length or width variability on metabolic power during walking. Random intercepts were adjusted for each participant using Eqn 1, where COV is the coefficient of variation of the step length or width during a trial:
(1)

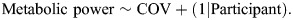


We used a likelihood ratio to test for significance between a model excluding step length or width variability and an alternative model that did not. A significant difference between the two models indicates that the inclusion of step variability improves model performance. Chi-squared (χ^2^) and significance values are reported. When evaluating step width and learning, we were only interested in comparing across projected conditions, without NP; therefore, we used a one-way repeated measures ANOVA to evaluate whether average step width was different across the projected conditions, and a two-way repeated measures ANOVA (condition by trial) to compare possible learning effects in the first and second attempt of each projected condition by testing whether metabolic power was lower when participants were re-exposed to a condition for the second time. When running our repeated measures analysis, we only compared across projected conditions to standardize the effects of walking over projections on step width and learning. For all statistical tests, significance was set at an alpha level of 0.05.

## RESULTS

**Fig. 2.**
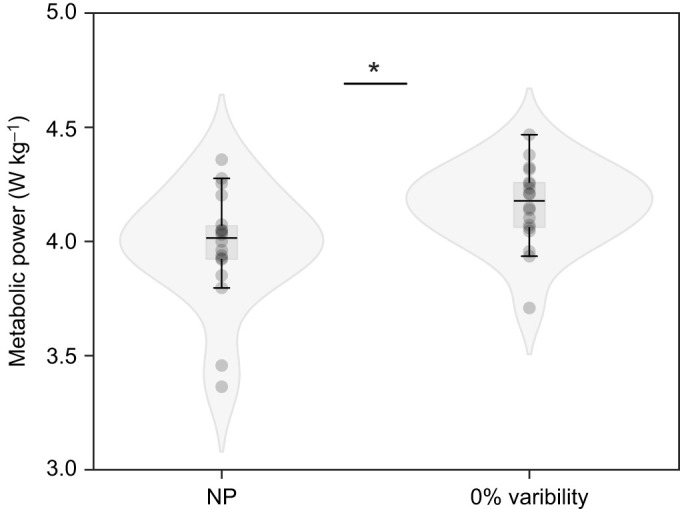
**Metabolic power in the no projection versus 0% variability condition.** Metabolic power (*n*=18) was lower for walking without any projected stepping stones (NP) than for walking over stepping stones projected without any step length variability (0%). We used a paired *t*-test to identify differences between conditions. Values are means±s.d., *P<0.05.

Metabolic power (3.98±0.25 versus 4.16±0.18 W kg^−1^; *P*=0.0002), step length variability (2.42±0.55% versus 3.17±0.64%; *P*=0.001) and step width (128±35 mm versus 152±28 mm; *P*=0.002) were different between NP and 0% conditions (Fig. 2). Therefore, when pooling the step length variability across trials, we excluded the NP condition (i.e. we included only data from the 0%, 5% and 10% step length variability trials in our model) to compare the effects of our intervention across similar levels of feedback. Our participants walked with 2.42% step length variability without projections and with 3.17±0.64 (0%), 4.38±0.98 (5%) and 6.94±1.07 (10%) step length variability in each of the three projected conditions. Although step width was greater in the 0% compared with the NP condition, it was unchanged across projected conditions (*P*=0.37).

**Fig. 3.**
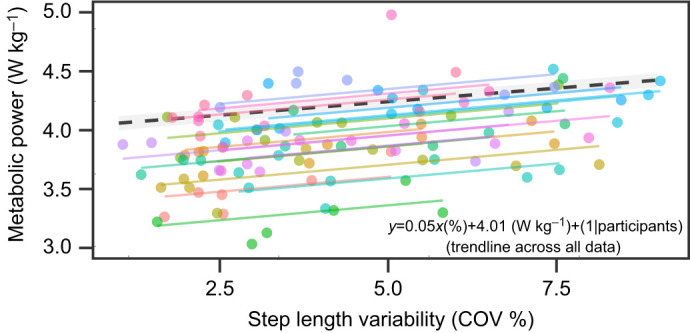
**Effect of step length variability on metabolic power.** For every percentage increase in step length variability, there was a 0.05 W kg^−1^ (1.1%) increase in metabolic power. The linear mixed effects model identified displays trends for each subject (*n*=18; colored lines) across trials.

Overall, metabolic power increased with increasing step length variability (χ²=9.41; *P*=0.002; Fig. 3), independent of changes in step width variability (χ²=2.82; *P*=0.09). For every 1% unit increase in step length variability, there was an increase in metabolic power of 0.05±0.01 W kg^−1^, or 1.1%. The linear mixed-effects model used for this analysis, *y*=0.05*x*(%)+4.01(W kg^−1^), revealed a significant fixed effect for COV (*P*<0.001). The marginal *R*^2^ was 0.10, indicating that 10% of the variance in metabolic power was attributed to step length variability, while the overall model *R*^2^ was 0.73, with the remaining variance explained by random effects (i.e. random intercepts for metabolic power).

To explore potential non-linear relationships, we also evaluated a model that included a squared term for step length variability. However, the additional term did not improve model performance compared with a linear fit (*P*=0.17) and was therefore not retained for further analysis. Results for average step width and learning effects showed no impact of condition on average step width (*P*=0.37), a main effect for trial on metabolic power (*P*=0.02), and no condition by trial interaction (*P*=0.27).

## DISCUSSION

In this study, we quantified the isolated effects of increased step length variability on the metabolic cost of walking. In line with our hypothesis, increases in step length variability resulted in increases in metabolic power. This increase in metabolic cost of walking from increased step length variability is substantially smaller than that observed in studies indirectly manipulating step length variability (O'Connor et al., 2012; Rock et al., 2018). By projecting virtual stepping stones during treadmill walking, we were able to keep gait parameters consistent across the three projection conditions included in our main analyses, with only changes in step length variability between conditions. By quantifying the relationship between the changes in step length variability and the changes in metabolic cost across these conditions, we were able to assess the isolated effects of step length variability, which are smaller than observed in studies where step length variability changed in response to indirect manipulation. While increased gait variability is often mentioned as a potential contributor to elevated metabolic cost of walking in older adults and people with neurological impairments (Christiansen et al., 2009; Osoba et al., 2019), our observations on young healthy adults suggest that the biomechanical consequences of walking with more variable step length only have a modest, though significant, effect on the metabolic cost of walking.

Our findings show a 1.1% increase in metabolic power per 1% increase in step length variability (Fig. 3) at a common walking speed of 1.20 m s^−1^. At this speed, a 1% change in step length variability increases metabolic power by 0.05 W kg^−1^. This increase is almost three times smaller than the 0.14 W kg^−1^ increase in metabolic power for every 1% increase in variability modeled by Rock et al. (2018). This difference is likely related to our direct manipulation of step length variability at a single, constant speed, eliminating the metabolic penalty of walking slower than preferred (Ralston, 1958). Additionally, the work of O'Connor et al. (2012) reported an increased metabolic power (5.9%) while walking with virtual visual flow perturbations (high-frequency medio-lateral rotations) and while step length variability increased by 27%, this was accompanied by a 65% increase in step width variability and 19% wider mean step width. This suggests that step width variability has a larger effect on metabolic power than step length variability. Indeed, walking with increased step width is known to increase metabolic cost (Donelan et al., 2001), and changes in step width are likely to have affected O'Connor et al.’s (2012) findings more than ours because step width was similar within our projected conditions.

Two biomechanical mechanisms can be expected to play a role in elevating the metabolic cost of walking with increased step length variability. First, adjusting to a closer stepping stone (i.e. reducing step length), increases metabolic cost because the legs move at a higher rate (Doke et al., 2005). Second, an adjustment to a farther stepping stone (i.e. increasing step length) increases the cost of lifting the center of mass over the point of collision (Donelan et al., 2001). Additional possible causes for the elevated metabolic cost of walking include an increase in muscle co-contraction, commonly observed with increased accuracy demands (Gribble et al., 2003), and the cost of consistently regulating steps across projected stepping stones, reducing the contribution of passive dynamics to walking (Wezenberg et al., 2011). Indeed, participants found it difficult to maintain the prescribed step length modifications. Step length variability was higher than projected for the 0% condition and lower for the 5% and 10% conditions. These additional mechanisms could help explain the differences in metabolic cost between walking with no projections (habitual) and walking with 0% step length variability projections that closely matched habitual gait characteristics (Fig. 2).

Our findings suggest that a small portion of the elevated metabolic costs observed in populations with increased gait variability (Hausdorff, 2005) is due to the biomechanical consequences of walking with more variable step length. Humans naturally walk with some degree of gait variability to regulate maintaining speed (Almarwani et al., 2016) and balance (Collins and Kuo, 2013). The larger gait variability in older adults and in people with neurological impairments could contribute to their increased metabolic cost of walking as compared with young (+8%; Martin et al., 1992) or neurologically healthy adults (+17–170%; Compagnat et al., 2020; Jeng et al., 2020; Rooney et al., 2022). For example, older adults walk with step length variabilities between 4% and 4.7% (Almarwani et al., 2016; Brach et al., 2010; James et al., 2020; Kang and Dingwell, 2008; Verlinden et al., 2013), which is on the lower end of the changes in variability we observed (2–10%), but 2.3% higher than the 2.4% observed in our NP condition. With our findings, we estimate that this 2.3% higher step length variability will increase the metabolic cost of walking by up to 2.8%. Similarly, people with neurological impairments have been reported to walk with step length variabilities that are approximately 2.5%, 3.0% and 6.0% larger than those of neurologically healthy controls for Parkinson's disease, multiple sclerosis and cerebellar ataxia, respectively (Buckley et al., 2018; Noh et al., 2020; Roemmich et al., 2012; Socie et al., 2013). Our data suggest that these increases in step length variability will increase the metabolic cost of walking by 2.8%, 3.3% and 6.6%, respectively, a change greater than observed from manipulating step length symmetry in people post-stroke (Nguyen et al., 2020). Overall, these modeled increases in the metabolic cost of walking from increased step length variability are likely to contribute minimally to the total increased metabolic cost (17–170%) of walking observed across these populations (Compagnat et al., 2020; Jeng et al., 2020; Martin et al., 1992; Rooney et al., 2022).

### Limitations and future directions

To ensure that the step length variations were perceivable to participants, we binned step lengths into 5% multiples, not exceeding 15% of their habitual step length. Normal walking does not contain such discretized step length variability. Although the perturbed conditions do not perfectly reflect real-world gait variability, they provide an upper limit for the increases in metabolic power in young healthy individuals. Most participants were unable to exactly match the discretized step length variabilities that were projected; while the use of 5% bins is different from real-world gait variability, participants still had trouble maintaining accuracy across the stepping stones without additional feedback. We were limited in identifying how participants altered their step length. In future research, it will be insightful to evaluate whether participants took larger or shorter steps when aiming for the stepping stone targets, as well as the frequency and magnitude of their errors. This could help in relating task performance to step length variability within a condition. While few participants were able to walk with 5% and 10% step length variability, 0% step length variability was unachievable – the lowest value for a single participant was 2%. This is in line with observations that walking inherently contains some variability in step length (Collins and Kuo, 2013), which might also be distinct between participants.

Participants did not receive feedback on how well they were performing during a trial. Feedback could have improved task performance (Shull et al., 2014) to better align with our variability conditions. We also cannot fully rule out that our increasingly variable visual environment did not partially contribute to differences in metabolic power. Furthermore, there was a main effect of trial on metabolic power, indicating that metabolic power was lower in the second trial of each condition, but as there was no condition by trial interaction, all conditions were affected equally, and our conclusions remain the same. To investigate whether co-contraction, to maintain accuracy (Gribble et al., 2003), contributes to the increased metabolic cost of walking with variable step lengths, future studies should quantify foot placement accuracy and muscle activity across similar virtual projections. Finally, to clarify the relationship between variability and gait deficiencies for populations already experiencing increased step length variability, further investigations should be conducted with these populations specifically.

### Conclusion

Older adults and those with people with neurological impairments walk with greater step length variability and increased metabolic power. In a population of healthy young adults, we found that metabolic power increases by approximately 1.1% (0.05 W kg^−1^) for every 1% increase in step length variability. Although most of our participants were unable to exactly match the projected conditions, our visual perturbation successfully increased step length variability and metabolic power across step variability conditions. The metabolic power per unit of variability was smaller than reported in previous work, indicating that step length variability plays a modest, albeit significant role in the metabolic cost of walking.
